# Characteristics and stability of consumer food-buying groups: the case of *food circles*

**DOI:** 10.1007/s41130-022-00172-4

**Published:** 2022-10-19

**Authors:** Kirsi Korhonen, Toivo Muilu

**Affiliations:** Natural Resources Institute Finland, Oulu, Finland

**Keywords:** Food-buying groups, Alternative food networks, Local food, Organic food, REKO rings, Finland

## Abstract

Demand for local and organic food has increased rapidly in Finland in the past two decades, as also have the wide variety of alternative food networks and food cooperatives (e.g. food-buying groups or food circles). However, the operating environment of traditional food-buying groups, aka *food circles* (*ruokapiiri*), has been experiencing changes as well. The supply of local and organic food in grocery stores has improved and new types of social media-based buying groups (REKO rings) have formed. This paper examines and evaluates the characteristics and stability of food circles in the Northern Ostrobothnia region in Finland by studying their structure and changes in their status over a 5-year period and reviewing their similarities and differences to REKO rings. An electronic survey and seven semi-structured interviews were implemented during the years 2013 and 2014. In addition, the status of the food circles was investigated in 2019 via email or phone, and REKO rings were studied through the literature. Food circles were usually seen as a functional way to purchase local and organic foodstuffs. Some members valued the high degree of traceability of shipments and face-to-face encounters with producers; for others, the primary motivation was overcoming issues of access and affordability. However, only two of the seven food circles interviewed were still operating in 2019. In addition, their level of activity had slowed down or transformed. Although most of the food circles were established by active consumers with more than just the intention to make local and organic food more available, it seemed that later on in operation most of the side activities faded and the groups failed to engage suitable people in the activity. Probably the most important difference between traditional food circles and modern REKO rings is the need to volunteer. In addition, social media-based REKO rings are better known on a mainstream level. On the other hand, our findings may indicate that while food-buying groups, as a form of alternative food networks, are a relatively new phenomenon in Finland, they might just be still applying their format.

## Introduction

The de-globalisation of food markets appears in the discourse of alternative food networks (AFN) (e.g. Goodman & Goodman, 2009; Renting et al., 2003; Sonnino & Marsden, 2006) which have been linked to broader concepts such as locality (e.g. Feagan, 2007; Hinrichs, 2003), sustainability (e.g. Forssell & Lankakoski, 2014; Stein & Santini, 2021) and short food supply chains (SFSC) (e.g. Kneafsey et al., 2013).

Some studies have attempted to identify consumer’s motivations and behaviour in AFNs (e.g. Mastronardi et al., 2019) and characterise the profile of the consumers who favour SFSCs (e.g. Dorottya, 2017) as well as their perceptions and preferences for local food (Feldmann & Hamm, 2015), as well as social inclusion in consumer food cooperatives (Fourat et al., 2020). In western societies particularly, many consumers want to know the origin of food; and they want it fresh and unprocessed (Brown et al., 2009; MacMillan Uribe et al., 2012; Saito & Saito, 2013). Moreover, food is often seen as an experience and it can be expected to be genuine and tasty (Finland’s 72^nd^ government 2013, p. 7).

According to Hendrickson and Heffernan (2002, p. 361), alternative food chain movements must be organised where the dominant system is vulnerable. While the supply of local food and particularly local organic food does not account for a large share of groceries in general, consumers are willing to find alternative ways to get what they want. One example of AFNs are different kinds of food cooperatives such as food-buying groups, although they are not a new phenomenon (Belasco, 2007). According to Belasco (2007), in the 1960s and early 1970s, food cooperatives in America were frequently constructed as a means of creating change through the everyday acts of food purchasing and distribution. In Finland, traditional food-buying groups, aka *food circles* (*ruokapiiri*), that have been longer in action are often centred on organic products, with additional emphases on locally grown food (Lamberg, 2009). According to Kallio (2018), food collectives in Finland remained fairly unknown until taking off in the 2010s, establishing themselves as one way to access and purchase locally farmed food on a regular basis.

Little et al. (2010) have studied food-buying groups and cooperative styles of purchasing in Europe, North America and Japan. They noted scant reference towards such groups in the alternative food and ethical consumption literatures. However, they observed that the relevant studies offer much—especially in terms of the historical context, future lessons for growth in the sector, and consumer motivation and ethics involved in buying groups. They (Little et al., 2010: 1798) suggest that: ‘collective purchasing groups may represent an important form of agri-food network and, crucially, may also offer greater room for consumer voice and action, capable of animating ethical consumption practice’. Buying groups act as enablers in the distribution of local and organic foods, and social and communitarian capital is also derived and generated through the process of collective action. According to Dedeurwaerdere et al. (2017), collective food-buying groups seek to bring about societal change by organising a protected space for learning and experimentation with lifestyle changes for sustainable food consumption and production practices.

Additionally, the development of food circles in Finland as sales venues for local food has stirred conversation about the communal nature of food acquisition (Kurunmäki et al., 2012). Food circles can be perceived as part of a communal economy, in which production and consumption take place voluntarily among people of a certain community and are based on a genuine will to participate in development and sharing (Forss & Kanninen, 2013, p. 7). The economy is thus a way to achieve social and ecological goals, and food circles are associated with consumer-citizenship.

The operating environment of food circles (see the definition by Lamberg, 2009 & Kallio, 2018 in Chapter *Food circles and REKOs in Finland*) in Finland has changed in recent years. Local and organic food have been on the political agenda, especially since 2010, and the supply of local and organic food in groceries has improved. In addition, a new type of Facebook-based model for selling and distributing local food regionally has been formed known as *REKO*, which stands for *REjäl KOnsumtion (fair consumption)* (see e.g. Yrkesakademin I Österbotten, 2016; Szymoniuk & Valtari, 2018; Kumar et al., 2021). REKO rings operate in closed Facebook groups, where a producer writes a Facebook post about his or her product and supply, and consumers order the goods by commenting directly below the post (Snellman, 2021).

The aim of this article is to examine and evaluate the characteristics and stability of food circles in Northern Finland by studying their structure and changes in their status over a 5-year period. We also review their similarities and differences to REKO rings, which seem to have largely replaced traditional food circles in the study region. There are not many studies regarding food-buying groups in Finland, however it seems that the interest to study them has evolved just during the recent years following the increasing popularity of REKO rings. On the other hand, traditional food circles can be seen having some kind of pioneer role in Finland, as they have been introducing the concept of collective buying, and making local and organic food more familiar to the consumer as well. The original data for this study was collected near the time when first REKO rings were born. The specificity of the timing sets an interesting framework to study the evolution and transformation of food-buying groups in Finland (especially Northern Finland).

Our evaluation in this study is partly based on three prominent characteristics identified by Little et al. (2010) that help to exemplify the reasons for the formation and growth of food-buying groups in general: (1) *the key drivers behind their formation*, (2) *the length of time they have been in operation* and (3) *the evolving legal status of the groups*. The concrete objectives of this study are formulated in the following research questions:

1. What are the characteristics of food circles in Northern Ostrobothnia?
2. What are the challenges of the traditional food circles in Northern Ostrobothnia and how stable do they appear?
3. How do traditional food circles compare to REKO rings?

After this introduction, the structure of this paper is as follows. First, we open with a definition of food-buying groups and food circles according to the literature and development of the local and organic food sector in Finland. Secondly, we introduce the materials and methods used in this study. Thirdly, we present the results, starting with the ideological background of food circle members and the issues related to food circle participation and operation and evaluate how they compare to REKO rings. Then, we discuss the current situation of the food circles included in the study such as their life cycle and challenges, and in this section also make some comparison to REKO rings. Finally, we present some concluding remarks concerning traditional food circles as well as food-buying groups on a more general level.

### Defining food-buying groups

In the USA, interest in food co-ops and food-buying clubs has been greatest during times of recession, as one of the goals of food circle activity has been a lower price level for food (Herrmann, 1991). However, according to Cox (1994), the motivations behind individual members’ decisions to join the cooperative movement are multi-varied. Ronco (1974, p. 34), for example, stated in the 1970s that variations on the collective theme were ‘endless’. Generally, the ideology behind the co-ops came first and the experiments in distributing and retailing came afterwards. According to Ronco (1974, p. 35): ‘The only ingredients necessary to start a food co-up are: a group of people, some space to put them in, some of their money to buy food, someone to sell them the food and some way for them to distribute the food back among the group. The specifics are open to much discussion among the groups themselves’.

Graham et al. (2002) and Gibson-Graham (2005) have suggested that buying groups can be viewed as a microcosm of a ‘diverse economy’, encompassing both corporate and not-for-profit, waged labour and payment-in-kind—as well as personal and communitarian gain. Little et al., (2010, p. 1799) have also argued that: ‘neglected elements of the economic process are crucially important in terms of understanding how food-buying groups and food cooperatives function’. The use of household and community spaces as well as labour practices helps reduce the overall cost of the products and increases access to goods because delivery locations can be selected by the groups themselves (Little et al., 2010, p. 1802). That is to say, buying groups are a potential mechanism for addressing issues both of access and of affordability. In addition, the collective distribution of goods from a central location encourages a social function by bringing people together from a certain community or neighbourhood.

However, according to Ronco (1974), as the number of members grow, food-buying groups face dilemmas between continuing growth and maintaining their founding principles. Kump and Fikar (2021) have recently evaluated the challenges of maintaining and diffusing grassroots innovations in alternative food networks by using a systems thinking approach. They came to the similar conclusion to Ronco (1974) that food cooperatives seem to have an ‘optimal size’, and when such systems become too large, there are negative feedback loops affecting motivational aspects of the users. They also noticed that the diffusion of alternative food networks into the mainstream may be achieved through replication and translation strategies, rather than scaling-up.

On the other hand, in their study on Belgian network of food-buying groups, Voedselteams (Food Teams, started in 1996), Zwart and Mathijs (2020) found that the routinisation and professionalisation of alternative practices may facilitate the participation of members who are willing to invest less time and effort in gaining access to food through an AFN. Zwart and Mathijs (2020) describe the development over time of Voedselteams and state how the growth of the organisation has resulted in notable routinisation and professionalisation of some of the practices. For example, a web-shop was developed to facilitate and professionalise the placing of orders, and transportation methods in some of the regions were professionalised and specialised.

Some other examples of successful food co-ops and food-buying groups in Europe are ‘Gruppi di acquisto solidale’ (Solidarity Purchase Groups or GAS) in Italy, which started in 1994 (e.g. Maestripieri, 2016), the French community supported agriculture movement ‘Associations pour le Maintien de l’Agriculture Paysanne’ (AMAP), with its first groups founded in 2001 (e.g. Lagane, 2015), and the community supported agriculture movement in the UK (begun in 2013) (CSAUK, 2022).

Little et al., (2010, pp. 1802, 1804–1806) see the phrase ‘buying group’ as an umbrella term, and despite diversity in the form, scale and function of buying groups, there are some prominent characteristics that help exemplify the reasons for their formation and growth.

The first characteristic involves *the key drivers behind their formation*. Although there might be multiple drivers behind the initiation of buying groups, the action of consumers appears to be a particularly powerful driving force behind their development. Consumers seem to have the ability to make creative interventions by forming new mechanisms to access local and organic foods.

The second characteristic is *the length of the time they have been in operation*; the historical context plays a significant part here. Lang and Gabriel (2005, p. 50) have estimated that ethical consumption started to come into its own in the 2000s, with a reaffirmation of ‘the moral dimension of consumer choice’. According to Little et al. (2010), many food-buying groups were initiated around 2000 with the objectives to support local farmers and promote more sustainable farming methods for instance.

The third characteristic is *the evolving legal status of the groups*. Efforts to formalise the structure go hand-in-glove with moves to increase the capacity of these groups to incorporate a greater number of members. These evolving structures typically take two forms: a cooperative model, managed on a collective basis through communal decision-making, or a coordinator model, where decisions are made on behalf of the group by a central coordinator (Little et al., 2010).

#### Food circles and REKOs in Finland

A simple Finnish definition of *a food circle* (*ruokapiiri*) is provided in a guide published in 1999 by the associations Friends of the Earth Finland (Maan ystävät ry) and the Finnish Biodynamic Association (Biodynaaminen yhdistys ry) (Airaksinen et al., 1999, p. 3): ‘A food circle is a group of people who buy their food together.’ Products can be purchased directly from farmers or ordered from special eco-shops or wholesale businesses (as in the case of imported products). Food purchased via the circle is portrayed as safe because the members of the circle know what it contains and how it has been produced. Voluntary labour and togetherness are an integral part of the circle. A recommended size for a food circle is approximately ten households, but according to Lamberg (2009), they have anything from dozens to hundreds of members. In her doctoral dissertation regarding Finnish food collectives, Kallio (2018) points out that food circles comprise thousands of household members and hundreds of farmers. Food circles are usually specialised in produce and other goods farmed and manufactured adhering to the principles of organic production (Lamberg, 2009). They can be registered or informal, but they are based on non-profit pursuits with as few intermediaries as possible. Orders are usually placed once a month and delivered to farmers, after which the products are distributed to the members on a pre-set distribution date.

According to Kallio (2018), food circles or food collectives can be found all around Finland with the densest concentration in the south of the country, specifically in the capital region. They may differ greatly on the basis of their size and location, as well as in terms of how they organise food procurement in practice. Kallio (2018, p. 46) states that: ‘Each food collective adapts to its surroundings and local conditions and ends up representing the needs and wants of its participants’.

Pro Ruokapiirit ry (‘Pro Food Circles’), established in 2014, is an umbrella organisation for food circles which provides counselling and training services and promotes food circle activities in Finland. According to Pro Ruokapiirit ry (2016), there were over 100 food collectives/food circles in Finland in 2016 (TEM 2019). However, it seems that in recent years Facebook-based REKO rings have replaced traditional food circles, at least to some extent.

According to REKO – Fair consumption since 2013 (Snellman, 2021), a recently published ‘e-book’ presenting the concept of REKO and how it has developed in less than a decade, there were 210 REKO rings with 4,000 producers, 435,000 members and 35 million euros in revenue in Finland in 2021. The first REKO rings were established in Finland in 2013 and the concept has also spread abroad, particularly to Sweden (see Gruvaeus & Dahlin, 2021) and Norway. In total there are over 600 REKO rings with more than 2 million members in 14 different countries. The operation of a REKO ring is described in the ‘e-book’ (Snellman, 2021, p. 22) as follows:

A REKO ring operates in a closed Facebook group. Producers and consumers who wish to participate in a REKO ring can apply to join the desired group. The admins, who are often a small group of key persons, accept the membership applications… A producer writes a Facebook post about his or her product and supply, and consumers order the goods by commenting directly below the post… The producer then delivers the pre-ordered products to the meeting place at the designated time for pick-up… The product pick-up usually takes place weekly or biweekly… All producers convene at the same place so that consumers can collect their orders from several producers at the same time.

The establisher of the REKO concept, Thomas Snellman, sees that Facebook is one of the reasons for the model’s success (Snellman, 2021, p. 18). There was no need to create a new tool because it was already there. The concept has also benefited greatly from media attention. Probably the most important difference, on a practical level, between REKO rings and traditional food circles is the need for volunteers.

### Development of local and organic food sectors in Finland

According to Enthoven and Van den Broeck (2021), there is no universal definition of local food systems (LFS), mainly because different interpretations of the ‘local’ scale exist. In the political sphere, LFS are defined differently across the world. In Finland, there are two commonly used definitions for local food. In 2000 the Finnish Working Group on Local Food (Maaseutupolitiikan yhteistyöryhmä, 2000) defined locally produced food to be the *production and consumption of food that uses raw materials and inputs of its own region of production, and promotes the economy and employment of the region*. The Central Union of Agricultural Producers and Forest Owners (MTK, 2011) defines local food as fresh, Finnish food produced as nearby as possible, with a known origin, producer and manufacturer.

According to Kallio (2018), markets for local food barely existed in the late 2000s in Finland and it was extremely difficult to access either local or organic food. However, local food has been on the political agenda, especially since 2010, and it is clearly recognised as a future growth sector in the National Local Food Programme (Finland’s 72nd government 2013) and government programmes (Valtioneuvoston kanslia, 2015, 2019). A government report on food policy (Valtioneuvosto, 2010) and the proposal for a national food strategy (Huomisen ruoka, 2010) promoted local and organic food as separate development areas. A new government report on food policy *Food2030* (Valtioneuvosto, 2017) was published in the spring of 2017. The report sets out the policy objectives and key priorities of the activities until 2030.

Since 2007, numerous projects have been implemented that cover issues related to local and organic food in Finland. According to a project listing compiled by Lappeenranta-Lahti University of Technology (LUT), there were at least 105 local and organic food-related projects implemented between 2007 and 2013 (LUT, 2014). In the past few years alone, the Rural Development Programme for Mainland Finland 2014–2020 has funded 91 development projects that mentioned ‘local food’ in their public descriptions (FFA 2020).

In addition, a nationwide ‘Local Food Coordination Project’ was implemented between June 2015–January 2018 which aimed at increasing and intensifying cooperation in the local food industry and enhancing the competitiveness of the sector through networking (RURAL.FI, 2020). Furthermore, since September 2018, similar action has been carried on in a nationwide ‘Food Sector Coordination Project’ (Aitojamakuja.fi, 2020).

During the past decade, several projects related to local food and the food sector have also been executed in the province of Northern Ostrobothnia. For instance, geographic information system (GIS)-based accessibility analyses were applied for analysing the potential for integral networking of local food production and transport companies (Korhonen et al., 2017). In addition, the possibilities of increasing the use of local food in institutional kitchens have been studied (Korhonen & Muilu, 2015). The Council of Oulu Region has also developed a strategy for the food sector in Northern Ostrobothnia that focuses on developing the local food industry (Vuorela, 2017).

## Materials and methods

**Fig. 1:**
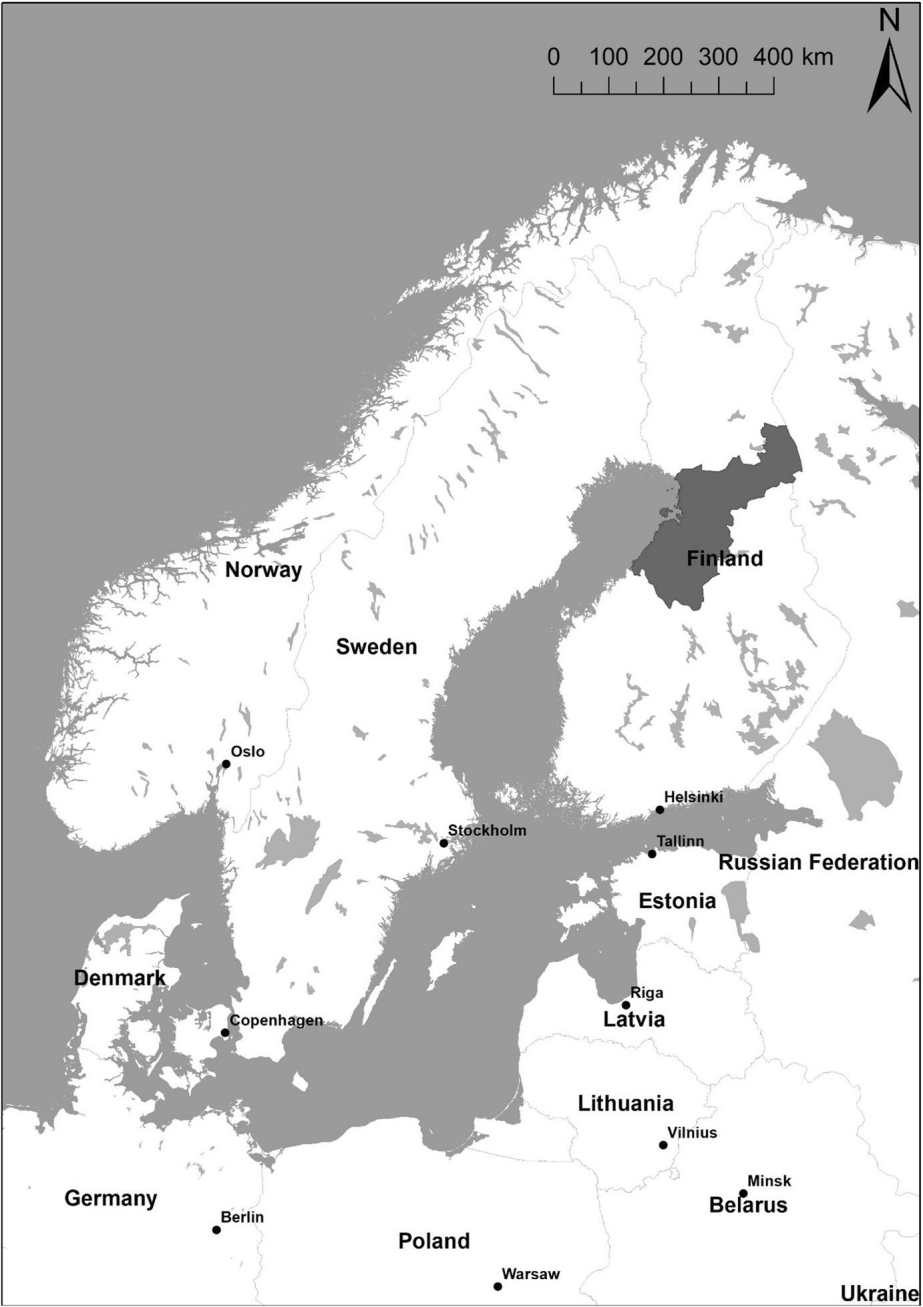
Location of the research area, Northern Ostrobothnia, in North Europe

This study was executed in the province of Northern Ostrobothnia, Finland (Fig. 1). This region has a population of 414,454 (in April 2021) and a total area of 45,851.98 km^2^ (2021). Almost half of the population live in the provincial centre in the city of Oulu, and the rest of the population are mostly located in the southern half of the region. Northern Ostrobothnia is a growing and developing region; its population is well educated and has the lowest average age (40.2 years) of any region in the country.

The food circles for this study were sought mainly via Internet search engines at the beginning of 2013. In addition, a notice was posted on the webpage of a rural communication project. This study covers a notable portion of Northern Ostrobothnian food circles and their members, while we reached 10 of 12 of the most well-known food circles and five other food circles. However, we must note that smaller food circles focused on a specific neighbourhood, for instance, are rather hard to find; we do not know their exact number in the region. To preserve anonymity, we do not mention the food circles involved in the study by name.

This study is based on the method of triangulation (e.g. Flick, 2007). Taking different perspectives on the issue under study and using several methods should produce knowledge at different levels (Flick, 2007). This goes beyond the knowledge made possible by one approach. The data gathering techniques selected for this research, considering traditional food circles, were surveys (questionnaires) and interviews, which were implemented in the RuokaGIS (*Accessibility of local and organic food in Northern Ostrobothnia*, executed in 10/2012–9/2014) project aiming to analyse local and organic food in geographic context and to develop its availability and access to markets in Northern Ostrobothnia. In addition, we used the literature for a comparative analysis of traditional food circles and REKO rings (which did not yet exist in the research area during the project). The research objectives of the project as well as previous Finnish studies related to local and organic food were taken into account in the design of the survey and interviews.

### Survey

According to Hirsjärvi et al. (2015), surveys or questionnaires can be used when gathering information about facts, behaviour and actions, knowledge, values and attitudes as well as beliefs, perceptions and opinions. The practice of using standardised questions in survey research is also based on the assumption that the responses will be given in a manner which allows the researcher to interpret and compare them (Järvinen, 2012, p. 143).

For this study, an electronic survey was sent to the organisers or other contact persons of 15 different food circles in Northern Ostrobothnia (all that we found) in the spring of 2013. As agreed with the contact persons, they distributed the survey link to the food circle members, so we do not know the exact number of survey recipients. The survey included mostly multiple-choice and Likert scale questions, that related to the definition, use and availability of local and organic food as well as to food circle activity overall. The survey data were derived using statistical descriptives, a nonparametric Kruskal Wallis Test, and crosstabulations.

A total of 15 food circles were represented in the responses to the survey; this included two inactive circles and three new circles that we had not heard of before. The survey gathered 119 answers (88% female and 12% male). Almost half of the respondents lived in the city of Oulu, the capital of the province. Half of the respondents represented those food circles that were also included in the interviews and about 14% did not mention by name any food circle that they participated or had participated in. We estimate, based on the number of food circle members according to the interviewees, that the survey responses cover about 13–19% of all households that participated in food circles in the study region during the time the data was collected. The backgrounds of the food circle members were highly variable (see Table 1); however, ‘the average member’ was somewhat similar to members identified in other studies related to food co-ops (e.g. Dorottya, 2017; Schifani & Migliore, 2011). It seems that food circles are mostly favoured by educated, working townswomen, who might voluntarily be on a specific diet (such as a vegetarian diet).

**Table 1:** Profile of the survey respondents (*n* = 119) in 2013

Age		Educational background		Situation in life	
	%		%		%
20–29 years	22.7	Comprehensive education	0.8	Working full-time	50.4
30–39 years	29.4	Upper secondary education	38.7	Working part-time	7.6
40–49 years	16.0	Lower tertiary education	30.3	Unemployed	7.6
50–59 years	21.8	Upper level of tertiary education	27.7	Student or pupil	16.8
60 years or older	10.1	Doctoral degree	2.5	Retired	7.6
				Took care of the household	0.8
				Parental leave	5.0
				Other reasons to be out of labour	4.2
Phase of life		Urban–rural classification*		Voluntarily on a specific diet**	
	%		%		%
Lived with their parent	0.8	Inner urban area	21.8	Yes	42.0
Lived alone	16.8	Outer urban area	32.8	No	58.0
Lived together with their partner	40.3	Peri-urban area	8.4		
Lived together with their partner and children	40.3	Local centres in rural areas	13.4		
Something else	1.7	Rural heartland areas	22.7		
		Sparsely populated rural areas	0.8		

^*^See SYKE’s (2019) urban–rural spatial classification of Finland

^**^Mainly vegetarian or low-carbohydrate diets

### Semi-structured interviews

Interviews can be used for instance when the research focuses on an unknown area and it is difficult for a researcher to know in advance the directions of answers, or when there is a willingness to clarify the answers available and when it is desirable to deepen the information available (Hirsjärvi et al., 2015). The semi-structured or non-formalised interview is neither a free discussion nor a highly structured questionnaire (Järvinen, 2012). In a semi-structured interview, the themes have been thought through, but in addition to them, specific questions have been prepared (Saaranen-Kauppinen & Puusniekka, 2006). A semi-structured interview is suitable for situations where it has been decided to obtain information on specific issues, and it is therefore not desired or necessary to give the interviewees very great freedoms in the interview situation. According to Eskola and Suoranta (1998), there is enough data when new cases no longer bring new information to the research problem, i.e. the material begins to repeat itself, so to speak.

Seven semi-structured interviews were implemented during the autumn 2013 and spring 2014. We interviewed the largest and most well-known food circles and a couple of smaller ones (some of them reached ten to twenty households and some up to 120 households) who agreed to be interviewed, to deepen the information gathered via the survey. Four of the food circles included in the interviews functioned or had functioned within the city of Oulu and three elsewhere in the Northern Ostrobothnia region. The interviewees were either frontmen (mostly women) or otherwise active members in food circles, which we regarded as key informants considering food circle activity (see Table 2). They all represented different food circles, and their ages varied from their early twenties to late forties. To preserve anonymity, we do not present their background information such as their age or gender per person. We asked them about issues related to food circle activity overall, their thoughts about local and organic food and their own reasons for participating in food circle activity. For support, interviewees were also asked to fill in a preliminary information form asking for more information on the activities in the food circle. The profile of the food circles reported in the interviews is presented in Table 3.

**Table 2:** Background information of the interviewees

	1	2	3	4	5	6	7
Role	Member, former member of the cooperative’s board	Active member (takes care of majority of the orders)	Frontman/	Frontman	Frontman/establisher	Frontman/	Frontman
Years active	9 years	Joined shortly after establishment (over 10 years)	establisher	About 3 years	Since beginning (about 3 years)	establisher	About 1.5 years

**Table 3:** Profile of the food circles interviewed in 2013 and 2014. Food circles 1 and 7 were still operating in 2019

	1	2	3	4	5	6	7
Establishers and operators	Established by few active consumers. Rotation of responsibility in operation. Registered cooperative	Established by few environmentally active consumers. Two frontmen. Informal	Established by two active people and operated by one frontman and few active members. Registered association	Established by few active people in the student union. Operated by two frontmen and few active members. Informal, subordinate to university	Established by few environmentally active consumers. Operated by one frontman with ‘assistant’. Informal	Established by few active people in political organisation. Operated by two frontmen. Informal	Born in contribution of a local food-related project. Operated by youth organisation
Legal status	Cooperative model, registered cooperative	Cooperative model	Coordinator model, registered association	Unclear	Coordinator model	Coordinator model	Coordinator model, youth organisation
Membership fee or equivalent	Yes	No	Yes	No	No	No	No, but youth organisation takes profit
Time of operation	In trade register since 1990	2001–? Not operating in 2019	2007–2017	2009–2012	2010–2015	2011–2012	2012– >
Estimated number of members/households	20	20	110	30–120	65	12	10
Number of members/households participating on order	Varies	Varies	–	30–80	25	5	1
Who can join in	Anyone but depends on the number of members which is limited	Anyone but new members are usually known by someone already involved	Anyone who lives around specific area. No limitations on the number of members	Students or staff from the university	Living or working in a particular neighbourhood. No limitations on the number of members	Anyone, no limitations	Anyone, no limitations
Regular space for distribution	Yes, semi-public	No	Yes, private (member’s home)	Yes, public	Yes, semi-public	Yes, private (member’s home)	Yes, public
Arranging of orders	Email	Email	Email	Online order form	Online order form	Email	Online order form, phone call or visit at the office/recycle shop
Frequency of orders	Depends on the product, fresh produce once a month	Depends on the product, fresh produce once a month	About three times per month	Once a month	Once a month	Once a month	Flexible
Emphasis on local or organic food	Local and organic	Local and organic, emphasis on organic	Local and organic, emphasis on organic	Local and organic, emphasis on organic	Local and organic	Local and organic, emphasis on organic	Local and organic, emphasis on local
Other activities	Joint meetings for members occasionally	Joint excursions in the beginning of operation	Joint meetings and lectures for members occasionally	‘Inner circle’ meetings			
Arranging of deliveries	Suppliers bring/other transport services	Suppliers bring/members pick-up/other transport services	Suppliers bring/frontman picks up/other transport services	Suppliers bring/frontmen pick-up/other transport services	Suppliers bring/other transport services	Suppliers bring	Suppliers bring
Additional information related to current (2019) status or ending	Not as busy as before. Availability of organic food in groceries has improved. Members are getting older, and their children have moved from home. Discovering new joint orders (flower bulbs, for instance)		Association closed down. Availability of local and organic food in groceries has improved	Too many subscribers in relation to organisers	Interest faded. Most took part in REKO rings	Several reasons for shutting down: logistics, not enough active members, too much driving, availability of local and organic food in groceries has improved	Small-scale action. Products (honey and flours) are sold only in ‘recycle shop’. Separate orders are not made. Give out surplus food from groceries as well

It must be noted that two of the seven food circles had already closed down in 2012, before the interviews. Nonetheless, we wanted to include them in the study, as they could offer valuable information and maybe give some indications on the characteristics of an unstable food circle. The status of the other five food circles was investigated at the beginning of 2019 via email or phone. The interview data were transcribed word for word and analysed manually by the means of thematising.

### Analysis of the REKO rings

The analysis of REKO rings is based on the literature, while they did not yet exist in the research area during the time the project was executed. The literature was searched via ScienceDirect and Google Scholar. There are not yet many international peer-reviewed papers regarding REKO rings, however, some theses have been done in recent years. The literature was examined using keywords regarding the same themes we dealt with for the traditional food circles.

## Characterising members and operating principles

In this chapter we first look at the ideological background of food circle members: how they define local and organic food and what their consumption habits are regarding those foods. After that we tackle the issues related to food circle participation and food circle operation and evaluate how REKO rings compare to traditional food circles.

### Defining local and organic food

According to Kotavaara et al. (2014), the definition of local food varies a little among different actors in the supply chain. However, the lack of middle men in the supply chain seems to be an essential feature of local food. There is no agreed distance limit for local food in Finland but in general discussion the distance limit varies usually from 50 to 100 km. On the other hand, local food commonly refers to products which are produced in the same province where they are used. The definition of organic food is largely based on regulations of the European Union (EUR-Lex, 2018) instead.

Since food circles serve as alternative or complementary channels for purchasing local and organic food, it is important to determine how the people using these distribution channels define those types of foods. For this study, food circle members were asked to define local food by its origin and distance limits. In addition, they evaluated five pre-given features that are commonly associated with local food and compared eleven different qualitative and production-related features between *Finnish food*, *local food* and *organic food*.

The geographical origin of local food was seen as follows: Northern Ostrobothnia or a neighbouring region (51%); the municipality respondent inhabits or a neighbouring one (43%); Finland (6%). The suggested average distance limit for local food was about 170 km (mode 100 km). It might be, that consumers who define the origin of local food more broadly taking into account the moderately long distances and climatic conditions (what products can be grown in these latitudes). They are realistic about the fact that not so many products can be (or are) produced in the vicinity of their place of residence.

The most important features associated with local food were, quite predictably, *there are as few elements in the supply chain as possible* and *the food has been produced in my area of residence*. On the other hand, the strengths of *local food* seemed to be that *it increases employment locally*, *its consumption feels morally right*, *it evokes trust* and that *further information on the products is available*. In comparison the strengths of organic food were that *it contains no traces of biocides*, *its consumption feels morally right*, and *it does not contain a lot of additives and preservatives*. Some benefits of *organic food* were also that it is perceived to be *environmentally friendly*, *trust-evoking, easy to recognize in a shop*, and that *further information on the products is available* and *it is not industrially produced*. *Finnish food* on the other hand scored highest on *availability* and *selection of products*.

### Thoughts about product information, purchasing habits and restrictions

As expected, local and organic food were found to be important to the respondents and they highly valued the information on food products in general, whether it regarded the origin, ingredients, or production methods (see Table 4). Over two-thirds of respondents reported buying local and organic food at least once a week. It is likely that some bought several different products and some only certain products. The average amount of spending on local and organic food per month was about 170 euros (*n* = 117). In comparison, the money spent per food circle order was approximately 50 euros (*n* = 92). According to these numbers, a less than a third of local and organic food purchases were realised through food circles.

**Table 4:** Opinions and procurement practices of food circle members on local and organic food in 2013. Total number of respondents is 119 unless otherwise mentioned

How important…*	Mean	SD	Which of the following best describes how you buy…	Local food	Organic food
local food is for you?	4.34	0.66		%	
organic food is for you?	4.38	0.82	I have tried sometimes but I usually don’t intentionally buy it	0.8	2.5
			I buy it occasionally a few times a year	2.5	5.0
			I buy it occasionally about once every couple of months	29.4	19.3
			I buy it regularly about once a week	42.0	25.2
			I buy it regularly several times a week	25.2	47.9
	Min	Max	Mean	SD	
	Euros				
How much money you spend on local and organic food per month? (*n* = 117)	10	700	172.44	142.69	
How much money you spend per food circle order? (*n* = 92)	10	250	49.45	38.69	
Acquisition channels I…	use at the moment to get local food	use at the moment to get organic food	would like to use to get local food	would like to use to get organic food	
	%				
Supermarket	54.6	59.7	29.4 (-25.2)	31.1 (-28.6)	
Farmer via food circle	47.9	61.3	35.3 (-12.6)	42.0 (-19.3)	
Corner shop	37.0	35.3	49.6 (+ 12.6)	42.9 (+ 7.6)	
Self-directed growing/gathering	33.6	32.8	27.7 (-5.9)	38.7 (+ 5.9)	
Direct purchase from the producer	25.2	26.1	28.6 (+ 3.4)	34.5 (+ 8.4)	
Market trader or alike	21.8	10.1	26.9 (+ 5.1)	26.9 (+ 16.8)	
Specialised retailer	11.8	17.6	26.9 (+ 15.1)	32.8 (+ 15.2)	
Online shop	4.2	15.1	10.9 (+ 6.7)	15.1 (-)	
Wholesaler	1.7	4.2	9.2 (+ 7.5)	10.1 (+ 5.9)	
How necessary do you regard the following information when purchasing food products (information on the label)?**				Mean	SD
Method of production				4.42	0.71
Place of production				4.65	0.59
Ingredients used in processed products				4.83	0.40
Place of origin of ingredients in processed products				4.72	0.52
Nutritional content				4.30	0.87
Willingness to pay for…compared to conventional food	A self-made meal		A meal in cafeteria or in a restaurant		
	Local food	Organic food	Local food	Organic food	
	**%**				
Less	0.0	0.0	0.0	0.0	
The same amount	15.13	5.04	10.92	8.40	
1–15% more	52.94	52.94	60.50	50.42	
16–30% more	25.21	32.77	23.53	35.29	
Over 30% more	6.72	9.24	5.04	5.88	
Restrictive factors on consumption of local and organic food***	Local food		Organic food		
	Mean	SD	Mean	SD	
Poor availability at the sales venue	3.33	1.050	3.08	1.054	
Other (*n* = 8)	3.13	1.553	3.50	1.414	
The sales venue is reachable with difficulty	2.76	1.247	2.62	1.221	
Price	2.54	1.177	2.88	1.202	
Difficult to identify	2.33	1.215	1.92	1.083	
Variability of quality	1.92	0.988	2.00	1.033	
Low degree of processing	1.76	1.095	1.73	1.087	

^*^Likert scale: 1 = not important at all; 5 = very important

^**^Likert scale: 1 = not necessary at all; 5 = very necessary

^***^Likert scale: 1 = does not restrict usage at all; 5 = fully prevents the usage

Other popular acquisition channels for local and organic food were mostly supermarkets and corner shops. Additionally, about 33% announced that they grow and gather food themselves. By ‘gathering,’ we mean berry and mushroom-picking as well as fishing and hunting, which are all relatively common leisure activities in Finland. It seemed that the respondents were willing to buy local and/or organic food clearly more often from corner shops, specialised retailers and market traders or the like, and less frequently from supermarkets or food circles. The last mentioned is in contradiction to the other response, which stated that about 33% of respondents would be willing to increase their amount of food purchases via food circles. This might reveal that even though the food circles are an interesting and appealing acquisition channel, they are not the first choice for the majority. After all, grocery stores particularly are usually an easier option for consumers due to their opening hours and because in any case people generally ‘must buy’ at least some basic products from them already whether that be toilet paper or salt and pepper for instance. It is only natural that today’s busy life(style) drives people to choose the simplest way to buy everyday food.

Most of the respondents were willing to pay more for local and organic food compared to conventional food. It might indicate that people are not primarily after lower prices when they are involved in food circle activity. In fact, those who bought local and organic food more often seemed to be willing to pay more for those products.

As earlier stated, according to the study by Little et al. (2010), some food-buying groups have the primary motivation to overcome the issues of access and affordability. Additionally, in our study, poor availability seemed to be a particularly restrictive factor on the consumption of local and organic food in general.

### Food circle participation and order activity

About 74% of the respondents participated in food circle activity at the time of completing the survey and 14% had associated with a food circle in the past (see more in Table 5). The average duration of food circle participation was 3.66 years (*n* = 85, members at the time of completing the survey), but the largest group, about 29%, was formed by those who had participated for at most 1 year. On average, the respondents ordered products via food circles almost 9 times a year, yet the largest group was formed by those who ordered products 1–5 times a year (37%).

**Table 5:** Food circle participation and order activity in numbers in 2013

Participation in food circle activity (*n* = 119)		Duration of food circle participation (*n* = 85)		Food circle orders per year (*n* = 101)	
	%		%		%
Currently participating	73.9	Max. 1 year	29.4	1–5 times	36.6
Previously participating	14.3	1.5 to 2 years	25.9	6–10 times	31.7
Never participated*	11.8	3 to 4 years	24.7	11–15 times	25.7
		Over 4 years	20.0	More than 15 times	5.9

^*^These members were most likely the customers of one producer who operated one food circle

It must be noted that some of the food circles were relatively new during the time the surveys were implemented, and about half of all respondents had participated in food circle activity for only a short period of time, so it might be that food circles had not yet evolved as regular food acquisition channels for them as for the other respondents. Additionally, according to the interviewees, it seemed that there were both regular subscribers who concentrated their food purchases to the food circles, and more occasionally participating ‘supporter’ members who purchased individual products every now and then through food circles.

### Food circle operating principles

Foodstuffs were usually ordered at least once a month via email (4 food circles) or some sort of online order form (3 food circles). One of the food circles operated in connection with a ‘recycling shop’ that served as a distribution base for deliveries but also had a few products for sale regardless of orders. All in all, six food circles had some specific spaces for distribution (either public, semi-public, or private spaces); with one group, the distribution space varied, depending on who organised the orders. Usually, suppliers delivered products straight to the distribution spaces or other transport services were used, but in three food circles the (most active) members also picked up some products from the producers. All food circles ordered both unprocessed and processed foodstuffs. Although the emphasis was generally on unprocessed foodstuffs, which are easy to deliver from the cold chain perspective and which do not require special storage facilities. Four food circles focused particularly on organic products and one on local products.

Despite the previously mentioned understanding that food circles might be appealing, to those looking for local and organic food at lower prices, the interviewees estimated that the price level of the products in the food circles was quite similar to the regular price for groceries. However, the interviewees reported that there were both more expensive and cheaper products, depending on suppliers and types of products.

### Food circles and REKOs—what’s the difference?

Although REKO rings were not the subject of the original project some preliminary comparisons to traditional food circles can be made on the basis of the existing literature. It seems that the most active members of REKO rings are quite similar in their background and ideology to the members of the traditional food circles. Some studies (Borgelin, 2017; Murphy, 2020) indicate that the most active members are older women (over 55 years). On the other hand, according to study made by Kolehmainen and Laitila (2016) based on observations of two REKO rings in the capital area, the consumers who were present at REKO pick-ups were between 20 to 50 years old, and there were evenly women and men. It is estimated that 190,000 Finns have shopped via REKO rings at some point (Snellman, 2021), so it is clear that more men are involved in these groups than in traditional food circles. There may also be regional differences in the membership of the groups as the studies above indicate. The reasons for purchasing food via REKO rings are also largely similar to the reasons for using traditional food circles. People would like to acquire fresh and high-quality food and support local producers and local economy at the same time (e.g. Kolehmainen & Laitila, 2016; Kantola, 2019).

Like traditional food circles, REKO rings also have both very active and quite passive members. In some REKO rings, the proportion of members actively purchasing food is estimated to be only couple of percent (Muikkula, 2017). According to Kolehmainen and Laitila (2016) some members would prefer to by local food from supermarkets. Additionally, the popularity of REKO rings seems to vary across the country (Rikkonen et al., 2017). Some rings appear more active than the others, and in some areas, the groups are perceived to have lost popularity after the initial rush, as they are schedule related.

The REKO ring running in the city of Oulu has product pick-ups biweekly (Pönkkö, 2019). Overall, the pick-ups in REKO rings are arranged clearly more frequently than in traditional food circles where the members typically order products once a month. On the other hand, another difference to traditional food circles is that REKO rings operate usually on a municipal level whereas traditional food circles usually operate in a neighbourhood. REKO rings offer quite similar products to traditional food circles, but the supply of fish and meat seems to be better. According to a study by Kiuru (2017), the price of organic meat and eggs, for instance, was felt to be cheaper via a REKO than at the grocery store. The quality of food compared to the price is perceived to be much better when purchasing from a REKO than in a grocery store as well. Additionally, in traditional food circles it seemed that there are both more expensive and cheaper products available.

According to Heidi Barman-Geust (2019), REKO consumers spend on average 564 euros per year for purchases via REKO. The number is up to a quarter larger than in food circles, where the sum would be about 450 euros when calculated from the average money spend per order and the average number of orders per year. This is probably a natural consequence of the fact that the product range in REKO rings seems to be slightly better than in traditional food circles, and more expensive products, such as meat and fish, are regularly available.

## Life cycle, challenges and development proposals

In this chapter, we look at the formation, functionality, cooperation possibilities and stability of food-buying groups. The results considering traditional food circles are based on the interview data we gathered and we will further compare them to REKO rings based on literature.

### Formation and growth

*The key drivers behind the formation* (see Little et al.’s (2010) theory in Chapter *Defining food-buying groups*) of almost all seven food circles were consumers. Six of them were established by one or more (environmentally) active consumers, and only one began in the context of a local food-related project. However, many establishers had some connections to different organisations and associations during the time of their formation. There were several reasons behind their formation in addition to the intention to make local and organic food better available. For instance, there was a willingness to promote local and organic food activities overall, to ‘avoid middlemen’ in the supply chain, to offer an opportunity to make environmentally friendly food purchases and to help people consider their consumption choices, respecting nature, encouraging ecological thinking and so on. This is consistent with the views of Dedeurwaerdere et al. (2017) and Hendrickson and Heffernan (2002) in their discussion of ‘seeking to bring about societal change’ and ‘creating a new kind of community’. In fact, some of the food circles arranged joint meetings, lectures, or excursions for the members—at least occasionally or at some point during their existence. However, it seemed that this type of action mostly faded and was marginal. Some of the interviewees thought that the need for these types of activities had decreased while most of the food circle members were already very ‘conscious consumers.’

*The length of time they have been in operation* varied from 1 to over 30 years. Most food circles in this study were initiated around 2010, which is congruent with Kallio’s (2018) study—the time when local food started to become a trend in Finland. However, the oldest and still-ongoing food circle had been in operation for over 30 years. The operation time of the ceased food circles varied from 1 to over 10 years.

*The evolving legal status* was not clear in all food circles, and they had different operating models. However, most showed more characteristics of the coordinator model than a communal model, while the decisions and practical implementation were mainly managed by one or two frontmen or a small group of active members. Two food circles seemed to fall more or less into the cooperative model category (with a strong rotation of responsibility in their operation). Most of the food circles were informal, but two of them had official rules and a subscription fee or equivalent.

Although different operating models were described as working during the time of interviews, the coordinative models seemed to be more laborious for the frontmen. This might have had effects, for instance, on the product selection and the frontman’s/frontmen’s ability to cope with the workload.

I have limited working time available for this, so I haven’t had time to invest so much, maybe we have quite a narrow range of products at the moment (Frontman of food circle 7, 2013).

Additionally, Kallio (2018) identified that some of the Finnish food collectives may appear to be an insecure and labour-intensive method for acquiring local and organic food. In fact, members of some collectives do get tired and question whether the amount of work they need to put into organising things is justified by the benefits of access to particular kinds of produce. According to Kallio (2018) and Ronco (1974), size plays a role in food collectives. In smaller collectives of 15 to 30 members, people are expected to become active participants who do not merely order the food, but also regularly volunteer to distribute it, while in larger collectives comprising more than 40 members, groups of more or less active participants are more likely to form (Kallio, 2018). The latter seemed to be a problem especially for one food circle in this study.

It did not demand enough responsibility from the people involved in the activity… it was supposed to be a community activity and doing things together… we organizers became like customer service representatives… (Frontman of food circle 4, 2013).

The REKO concept is a good example of a grassroots innovation in AFNs which have made small-scale food production visible to the mainstream through replication and translation strategies, which is more efficient than scaling-up according to Kump and Fikar (2021). REKO rings do not offer any ‘side hustles’ as some traditional food circles might do, instead the focus is on acquiring local and organic food.

REKO rings are seen mostly easy from the viewpoint of consumers, while no commitments or fees are required (e.g. Kantola, 2019; Pönkkö, 2019). However, a few volunteers are required as well. They administrate the Facebook group and organise producers in their area and direct traffic during pick-ups (Köngäs & Upola, 2019). The administrators also decide which actors can join the ring, so basically the activity is not open to everyone (Kolehmainen & Laitila, 2016). On the other hand, the administrators may change over in a relatively short period of time. According to a study by Pietikäinen (2018), one group of administrators serves on average for about a year at a time. However, the amount of work for administrators is clearly lower than in traditional food circles.

### A functional and ecological acquisition channel?

All the interviewees said that food circles, for them, were primarily a way to purchase local and organic foodstuffs; almost all experienced it to be a rational and functional acquisition channel. Food circles seemed to be successful in making specific products available for interested consumers, and finding new producers and suppliers. However, even food circles sometimes encounter problems with availability. The limitations of local production and small organic yields were noted by some of the interviewees. When the supply is limited, the food circles must be satisfied with what is available. Additionally, if the number of orders was not sufficiently large, sometimes the producers would not make the delivery.

One of the challenges we identified was that there was a set of more occasionally participating ‘supporter’ members involved in food circles, as shortly discussed earlier. Whether or not it was a consequence of unclear legal status or poor management, it had practical effects. The average number and monetary size of orders were relatively low—on average, about one third of local and organic food bought by households was bought via food circles when measured in euros. In addition, some of the interviewees stated that they (frontmen) themselves sometimes made large orders so that the orders could be fulfilled. In the case of one food circle, it seemed that not even all the establishers were actively participating in orders.

… Those who were actively setting it up in the beginning, did not order every time… (Frontman/establisher of food circle 6, 2013).

Some of the interviewees considered that food circles offered an opportunity to make ecological consumption choices, and many had wishes related to reducing the food transportation mileage and logistics emissions by shortening the food chain. However, not all interviewees (or food circle members) considered the ecology of food circles to be obvious; they pondered the rationality of food circle logistics as the members lived in a quite scattered fashion and the number of ordered goods per person might have been rather small.

I would somehow like to calculate more about whether the logistics makes sense …it has opportunities, if you can scale that activity upwards… (Frontman/establisher of food circle 5, 2013).

What annoyed me the most was the amount of driving, which was quite disproportionate compared to the amount of goods ordered… (Frontman/establisher of food circle 6, 2013)*.*

On the other hand, there have been many studies about the sustainability of local food and alternative food networks (e.g. Brunori et al., 2016; Edward-Jones et al., 2008; Forssell, 2017) regarding food miles particularly, and they have shown that transportation is a minor source of carbon emissions. However, some consumers might consider the efficiency of the overall food logistics. According to the survey, some of the food circle members carried out joint transportation with other members while picking up the deliveries.

In REKO rings the discussion about the challenges considers producers, in particular. According to Rikkonen et al. (2017), there are some challenges especially if a producer is involved in more than one ring (which mostly is the case). Assembling the orders is laborious and one might need to drive long distances to different REKO pick-ups, so there must be enough sales to make it profitable. Some other downsides identified in REKO rings are high prices and impracticality regarding the ambiguity of placing and retrieving orders, the time and planning required, the limited product range and payment methods, as well as the use of Facebook (Kantola, 2019). In the study made by Köngäs and Upola (2019) high prices were found to be the second most important reason for not using a REKO ring. The main reason was that the delivery times were not suitable. However, in general the prices were seen to be moderate and affordable.

On the other hand, because REKO rings operate via Facebook a user account is usually needed. According to Pietikäinen (2018), some older people who do not use Facebook, know the place and schedule of pick-ups, and might come and make purchases without placing pre-orders. Some producers also deliver products outside the REKO-organised pick-ups, but the information reaches these customers more slowly via printed product lists, e-mail or phone, for instance, which makes it more laborious for the producer. It seems that for the potential consumer the challenge in accessibility of traditional food circles is that they are difficult to find, whereas REKO rings usually come with technological requirements.

### Forms of cooperation

At the beginning of the study, we noticed that many traditional food circles were hard to find, and some of them had limitations on who could join. One was established for students and staff of a university, and a couple of them were operating in a specific neighbourhood or area. Additionally, in those food circles that basically had ‘open access’, the new members were usually known by someone already involved in the group. It remains unknown whether this also plays a role in the stability of some food circles. When asking about the development proposals and possibilities, some of the interviewees stated that food circles should be location based. However, four interviewees thought that food circles would benefit from a cooperation model or network on a higher level, and only one brought up development proposals that were clearly linked with the founding ideology of many food circles: to promote local and organic food activities overall.

I have a vision that food circles would be neighbourhood based and they would have some kind of co-operation model – it would be easier to operate if there was some common approach and certainly it would also be easier for the food circle organisers with ready-made concepts and tools. (Frontman/establisher of food circle 5, 2013).

In the case of REKO rings, many producers sell their products through several rings. The pick-ups are often arranged so that they are on the same day in neighbouring municipalities for instance. Otherwise, different rings operate independently. However, according to Snellman (2021, p. 69), one unexpected outcome of the REKO concept is *that the producers now have colleagues they meet regularly at various REKO drop-offs in the region. This has also created a distribution network with producers who buy from each other and help each other to deliver products.* It is interesting to see whether this could be the long-awaited boost for logistical cooperation between small producers also outside REKO rings, as the necessity for cooperation has long been called for (see Piilo 2007).

In addition, there exists some cooperation between a local food web-shop and REKO rings in Northern Ostrobothnia. At least some products can be ordered via the web-shop and retrieved from REKO pick-ups. It will be interesting to see how this function develops and compares to other cases. For instance, the study by Zwart and Mathijs (2020) noted how the routinisation of Belgian Voedselteams evolved into a web-shop.

### Stability of traditional food circles and future prospects for REKO rings

Two of the seven food circles interviewed in 2013 and 2014 were still operating in 2019 (see Table 3). One has been operating for over 30 years and the other for about 7 years. However, five food circles had closed down (two of them already in 2012). The reasons behind this were multitudinous, but the improved availability of local and organic food in general seemed to be one of the most common factors. The other given reasons included issues such as: the food circle had too many subscribers in relation to organisers and the workload for the organisers had expanded too much; logistical problems such as too much driving; lack of active members; faded interest; members took part in REKO rings; or the association behind the operation of the food circle closed down.

I achieved what I wanted by other means, that is, these products came into stores… on the other hand, the food circle had disadvantages that I had not thought about in advance, that is, this absurd driving and ‘supporting the food circle for image-related reasons,’ (Frontman/establisher of food circle 6, 2013).

The activity ceased as interest waned. Probably a part of it is that a large part of the food circle members joined the new REKO ring (Frontman/establisher of food circle 5, 2019).

The food circles still operating in 2019 had discovered new products and additional activities such as joint orders for flower bulbs and small-scale redistribution of surplus food. However, their level of activity also seemed to be slowing down or had transformed. The representatives of these two food circles further stated that the need for food-buying groups had decreased in Northern Ostrobothnia in recent years particularly due to the improved availability of local and organic products in grocery stores and the establishment of REKO rings. In addition, the active members of one food circle were getting older, and their children had moved away from home. The operation principles of the other food circle (Food circle 7 in Table 3) were comparable to retail operations, while separate orders were not made anymore.

The activity is quieter than before because the availability of organic food in stores is good. Our members have also become older, and their children have left home. We order wholesale orders and there does not seem to be a need for large quantities. Maybe even cooking and baking has decreased… (Member/former member of the cooperative’s board of food circle 1, 2019).

As mentioned in Chapter *Food circles and REKOs in Finland*, Pro Ruokapiirit ry, an umbrella organisation for food circles, was established shortly after our first round of our interviews. In 2016, they published a guide for new food circles. It remains unknown whether the food circles in this study would have benefited from this type of organisation. We did not find any contact information for the organisation and do not know if they are still active or not. This also supports the assumption that REKO rings have indeed replaced traditional food circles to some extent.

When it comes to REKO rings, their number continues to grow globally, however, development in Finland has stabilised (Snellman, 2021, p. 78). This is partly due to greater supply of locally produced food in grocery stores but also because of communication. Additionally, the establisher of the concept, Thomas Snellman, questions how long Facebook will retain its popularity, and how the REKO rings can then reach new, younger customer groups. Some actors are already looking for alternatives but none have come up that preserve *the essence and soul of interaction as well as Facebook groups—not to mention that it would need to be free-of-charge and devoid of a third party*. Pietikäinen (2018) also noticed in her study that producers could see that REKO rings might work without Facebook but not as efficiently.

Furthermore, Samsioe and Fuentes (2021) stated in their study regarding digital food platforms and their role in sustainable food consumption (includes a case study of six REKO rings in Sweden) that there seems to be no guarantee that new modes of food provisioning would become routinised over time. They might face challenges that are linked to the structure of the new routine established and its position in the nexus of everyday practices. The challenge in REKO rings particularly regards the development of a new shopping routine with a novel stand-alone temporality. Consumers need to remember to check their Facebook groups for new offers, place orders, pay for them and the pick them up every other week, for instance. According to Samsioe and Fuentes (2021), it was not uncommon for consumers to forget to place an order or forget to pick them up when the pick-up event came around.

Here again the question arises of how much extra effort average consumers are willing to put in and to create new routines to get food for their daily lives when easier alternatives are available. Enthoven and Broeck (2021) concluded in their review article that despite the active promotion of LFS in North America and Europe, it remains a niche market that has not substantially grown over the past years.

## Conclusions

The aim of this article was to examine and evaluate the characteristics and stability of *food circles* (*ruokapiiri*), traditional food-buying groups in Northern Finland by studying their structure and changes in their status over a 5-year period (2013–2019) and reviewing their similarities and differences to new REKO rings. This study reached a notable portion of the most well-known Northern Ostrobothnian food circles and their members via an electronic survey and interviews. The study was carried out at a time when development in local and organic food sectors was extremely rapid and the popularity of social media-based REKO rings had exploded.

First, we were interested in the characteristics of food circles in Northern Ostrobothnia. It seemed that people with different backgrounds had at least some interest in food circles. However, ‘the average member’ was somewhat alike members identified in other studies related to food co-ops—educated, working townswomen. The most important factor restricting the consumption of local and organic food was availability. Even though the traceability and face-to-face encounters with producers were highly valued in some food circles, the traditional and easy-access acquisition channels such as supermarkets and corner shops were also favoured ways to purchase local and organic products. Food circle members ordered products relatively rarely, usually once a month, which did not really show in the average monetary size of orders. Food circles took advantage of a variety of sales channels, from producers to wholesalers, and were flexibly looking for new opportunities without being dependent on specific suppliers.

Secondly, we wanted to identify the challenges of the traditional food circles in Northern Ostrobothnia and evaluate their stability. Food circles were usually seen as a functional way to purchase local and organic foodstuffs and offered an opportunity to make ecological choices. However, only two of the seven food circles interviewed were still operating in 2019. Among those who ceased operations were also food circles that had been in operation for over a decade. Food circles sometimes faced challenges such as a lack of active members and increased driving, which contradicted their founding principles such as sustainability. Additionally, probably behind these were poor management and their unclear legal status, and we believe that official rules and subscription fees or the equivalent might have helped to engage more suitable and active members — as the other one of the two still operating food circles had these. On the other hand, if a food circle operates on a rather ‘closed’ basis, it might even be expected that at some point its life cycle comes to an end. Members of the group grow out of the demand for such action for one reason or another, and in fact, the food circles under this study had a relatively low transition in their member base. We have not actively followed whether new traditional food circles have been established in the study region.

Thirdly, we were interested in how traditional food circles compare to the newly formed REKO rings, while they seem to have largely replaced traditional food circles in the study region. According to our findings, it seems that traditional food circles and modern REKO rings do not have many differences on a practical level. The most active members are quite similar in their background. However, the REKO rings are better known on a mainstream level and buying through them is at least easy to try as they are social media-based. Pick-ups in REKO rings are more frequent and the product range also seems to be wider on average, but of course, this depends on the ring in question. While REKO rings operate geographically in larger areas, it seems that the involvement of its members is not as important, as there are enough subscribers in any case. Additionally, REKO rings face some challenges, but they are more related to the workload of producers, and they do not depend on volunteers, which is at the heart of the activity of traditional food circles. On the other hand, the concept is based directly on the Facebook platform and its continuity is questioned.

The REKO ring in the city of Oulu is reported as one of the most active REKO rings in Finland. The demand for these kinds of groups might indicate that local and organic food is still scarcely available in Northern Ostrobothnia (partly due to the limited range of local agricultural products available), or other aspects such as the social value and face-to-face interaction without middlemen may be the driving force. We must also note that buying groups, as a form of alternative food networks, are a relatively new phenomenon in Finland, and they might just be applying their format in a time when the development on the food sector is rapid and information spreads quickly. The geographical conditions, particularly the number and density of the population in Northern Ostrobothnia are also totally different from the capital area in Finland, where traditional food circles are more popular.

It must also be noted that conventional retail businesses seem to have responded rather quickly to consumers’ increased demand for local and organic food. The information on food products in general was highly valued by the members of food circles, and in fact, nowadays it is common in Finland that even ‘bulk products’ have very accurate product information on their packages (e.g. the name of the farm the product has originated from). Additionally, in supermarkets and corner shops there might be labels on the store shelves that state ‘local product’ or ‘organic’. Online sales and home deliveries have also made acquiring food easier, and the use of such services has dramatically increased during the time of the COVID-19 pandemic. It will be interesting to see whether the demand for them solidifies its position in the long run.

The improved availability of local and organic food in conventional acquisition channels, as well as improved traceability overall, has certainly reduced the need for food-buying groups for many consumers. Both in traditional food circles and REKO rings seemed to have members who prefer to by local food from supermarkets. This fits well with Little et al.’s (2010) understanding that for some members the primary motivation for joining a food circle or corresponding participation is to overcome issues of access and affordability. Time will tell whether some other motivations raise and trigger new groups to be established in the region in the future or how the current ones will evolve. It is likely that access cannot be the only driving force for a stable food circle activity. Based on our study, viable action in food-buying groups most of all probably requires unambiguous coordination, and clear division of responsibilities as well as participants who match the ideology of the group. All these points came true in the food circle that was still operating in a quite ‘traditional manner’. Although it seems that the functionality and success of traditional food circles as such today is questionable, in any case it can be stated that they have had an important pioneer role in Finland, as they have been introducing the concept of collective buying, and making local and organic food more familiar to the consumer as well.

## Data Availability

The data is not available for other studies or purposes, since no consentments regarding sharing of the data has been requested or obtained from the target group of the study (GDPR).
